# Graph based fusion of miRNA and mRNA expression data improves clinical outcome prediction in prostate cancer

**DOI:** 10.1186/1471-2105-12-488

**Published:** 2011-12-21

**Authors:** Stephan Gade, Christine Porzelius, Maria Fälth, Jan C Brase, Daniela Wuttig, Ruprecht Kuner, Harald Binder, Holger Sültmann, Tim Beißbarth

**Affiliations:** 1 German Cancer Research Center, Cancer Genome Research, Im Neuenheimer Feld 460, 69120 Heidelberg, Germany; 2 Institute of Medical Biometry and Medical Informatics, University Medical Center Freiburg, 79104 Freiburg, Germany; 3 University Medical Center Göttingen, Medical Statistics, 37099 Göttingen, Germany; 4 Institute of Medical Biometry, Epidemiology and Informatics (IMBEI), Working Group Medical Biometry, University Medical Center Johannes Gutenberg University Mainz, 55101 Mainz, Germany

## Abstract

**Background:**

One of the main goals in cancer studies including high-throughput microRNA (miRNA) and mRNA data is to find and assess prognostic signatures capable of predicting clinical outcome. Both mRNA and miRNA expression changes in cancer diseases are described to reflect clinical characteristics like staging and prognosis. Furthermore, miRNA abundance can directly affect target transcripts and translation in tumor cells. Prediction models are trained to identify either mRNA or miRNA signatures for patient stratification. With the increasing number of microarray studies collecting mRNA and miRNA from the same patient cohort there is a need for statistical methods to integrate or fuse both kinds of data into one prediction model in order to find a combined signature that improves the prediction.

**Results:**

Here, we propose a new method to fuse miRNA and mRNA data into one prediction model. Since miRNAs are known regulators of mRNAs we used the correlations between them as well as the target prediction information to build a bipartite graph representing the relations between miRNAs and mRNAs. This graph was used to guide the feature selection in order to improve the prediction. The method is illustrated on a prostate cancer data set comprising 98 patient samples with miRNA and mRNA expression data. The biochemical relapse was used as clinical endpoint. It could be shown that the bipartite graph in combination with both data sets could improve prediction performance as well as the stability of the feature selection.

**Conclusions:**

Fusion of mRNA and miRNA expression data into one prediction model improves clinical outcome prediction in terms of prediction error and stable feature selection. The R source code of the proposed method is available in the supplement.

## Background

High throughput techniques, such as gene expression arrays, have made it possible to identify biomarkers and gene signatures for a wide range of diseases. For breast cancer several gene signatures have been proven to have a prognostic value [1-3]. Based on these, multigene tests like MammaPrint and Oncotype DX have found their way into clinical practice [4]. However, the efforts in using gene expression data to stratify cancer patients unraveled general limitations. Prognostic or predictive signatures are often restricted to a subset of patients which meet specific inclusion criteria like epidemiological, histopathological and clinical characteristics. Furthermore, gene expression data alone often did not reflect robust molecular subtypes in other cancer entities. For example in prostate cancer, one of the most frequent cancer types among men [5], the robust molecular diagnosis of a clinical relevant disease is still a challenge [6].

Genome scale experiments measure thousands up to millions of features. To be able to build clinical prediction models with these data, methodology from the field of machine learning is applied. Popular methods include SVM [7], Random Forests [8], and certain boosting approaches [9]. A particular challenge is the high number of features in the training data, especially if the correlation structure among the measured features is unknown. Therefore, the training results often remain unsatisfactory. In the past, integration of other sources of data that lead to an improved feature selection and thus to a better generalization of the prediction model has been discussed. Recent methods have integrated estimates of the correlation structure of the data based on prior information represented as graphs. The graph was gained from biological knowledge on interactions between genes or membership of genes to common pathways [10-15]. Other methods have integrated different kind of omics data [16]. When integrating data from different levels, properties and scales have to be taken into account as well as the relations between the different types of features.

Here, we propose a new method to fuse gene expression data with microRNA (miRNA) expression data into one risk prediction model. miRNAs are small, around 22 base pairs long, non-coding RNAs that regulate gene expression post-transcriptionally. By sequence mediated binding of the miRNA to its target, the translational process is blocked or the mRNA is predisposed to degradation. Deregulation of miRNAs has been linked to development and progression of several tumor entities including prostate cancer [17-20]. Because of their regulatory nature, the primary targets of a miRNA are of particular interest. Since experimentally validated targets are rare, target prediction algorithms are an important source of knowledge when dealing with miRNA expression data. Several algorithms and databases for miRNA target predictions have been established in the last years including e.g. miRanda [21], TargetScan [22], and PicTar [23].

Our new method uses a bipartite graph combining correlations between miRNA and gene expression data, and target prediction information. This gave rise to better prediction results compared to the single data sets in a prostate cancer data set encompassing 98 tumor samples.

The manuscript is organized as follows. The first section describes the general setup including high-dimensional time-to-event data and the measure of prediction error as well as the prediction methods. In the results part the final workflow is explained in detail and the performance on the prostate cancer data set is shown. Comparisons with two other prediction methods suited for time-to-event data are shown as well. The manuscript closes with a discussion and conclusion.

## Methods

### Setup

#### High dimensional time-to-event data

Time-to-event data, such as survival data, is typically modeled using the Cox proportional hazards model [24] of the form

(1) $$h\left(t|{\text{x}}_{\text{i}}\right)=h_{0}\left(t\right)\text{exp}\left(\eta_{i}\right)$$

with an unspecified baseline hazard *h*_0_(*t*) and a linear predictor

(2) $$\eta_{i}={{\text{x}}_{\text{i}}}^{T}\beta$$

Usually, observations are of the form (*t*_1_, *δ*_1_, **x_1_**), ..., (*t_n_, δ_n_*, **x_n_**) where *t_i _*is an observed time, *δ_i _*a censoring indicator (1 indicates an event while 0 indicates censoring), and **x_i _**= (*x*_1_, ..., *x_p_*) a feature vector. The Cox model describes the instantaneous risk of having an event at a given time point *t*. In a high-dimensional setting **x_i _**and thus *β *comprises several thousands of features, most of them irrelevant for predicting *h*(*t*|**x**). Therefore, it is reasonable to assume most of the entries in *β *to be 0 and methods with an implicit feature selection are preferable.

#### Prediction error curves and IPEC

**Figure 1:**
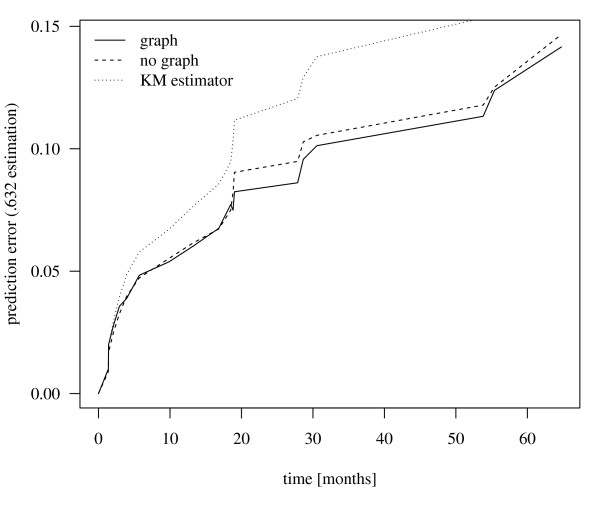
**Comparison of Prediction Error Curves**. The figure shows the prediction error curves of CoxBoost models trained on the mRNA and miRNA data. The prediction model was trained with and without the bipartite graph describing the relations between the features. The incorporation of the graph resulted in a reduction of the prediction error. The .632 estimation of the prediction error was used in this plot, averaging over the 500 bootstrap samples. As a reference the prediction error of the Kaplan-Meier estimator is shown.

The estimation of the Cox parameter vector $\hat{\beta }$ can be used to obtain a risk prediction

(3) $$\hat{r}\left(t|{\text{x}}_{\text{i}}\right)=\text{exp}\left(-{Ĥ}_{0}\left(t\right)\text{exp}\left({{\text{x}}_{\text{i}}}^{T}\hat{\beta }\right)\right)$$

with the Breslow estimator of the cumulative baseline hazard ${Ĥ}_{0}\left(t\right)=\int_{0}^{t}h_{0}\left(s\right)ds$. The predicted probability $\hat{r}\left(t|{\text{x}}_{\text{i}}\right)$ of still being event-free at time *t *can be seen as predicting the true status *I*(*t_i _> t*). To assess the quality of these predictions the Brier score

(4) $$BS\left(t\right)=E\left[\frac{1}{n}{\overset{n}{\underset{i=1}{\sum }}\left(I\left(t_{i}>t\right)--\hat{r}\left(t|{\text{x}}_{\text{i}}\right)\right)}^{2}\right]$$

can be used [25], describing the average discrepancy between the event states and the model predictions. Due to censoring, inverse probability of censoring weights have to be used to obtain consistent estimates of (4). By tracking this empirical version of the Brier over time, prediction error curve estimates are obtained:

(5) $$\begin{array}{ccc} PEC\left(t\right)=\frac{1}{n}\overset{n}{\underset{i=1}{\sum }}\left[\hat{r}{\left(t|{\text{x}}_{\text{i}}\right)}^{2}I\left(t_{i}\leq t,\delta_{i}=1\right)\frac{1}{Ĝ\left(t_{i}\right)}\right) & & \\ & \left(+{\left(1-\hat{r}\left(t|{\text{x}}_{\text{i}}\right)\right)}^{2}I\left(t_{i}>t\right)\frac{1}{Ĝ\left(t\right)}\right] \end{array}$$

where  is the Kaplan-Meier [26] estimate for the censoring distribution *G *[25] (cf. Figure 1). By integration over time the integrated prediction error curve (IPEC) is obtained. Here the R-package peperr [27,28] was used for assessment of model predictions.

#### Estimation of prediction error

To estimate the prediction performance (and compare it among different models) for new patients without the need to set aside test data the .632 bootstrap estimator [29] was used. For every bootstrap sample the .632 estimator of the prediction error curve was calculated leading to the IPEC.

### Prediction Methods

#### Boosting

Boosting belongs to the class of ensemble learners. The basic principle of boosting is the weighted combination of several weak classifiers in order to build one strong classifier [9]. This is equal to iteratively fit an additive model in function space by minimizing a loss function [30].

Componentwise likelihood-based boosting [31] uses a penalized log-likelihood criterion to fit the objective function. In every step only one element of the parameter vector *β *is updated which in fact is an implicit feature selection and results in sparse fits. Since the objective function is rather general the idea can be extended to high-dimensional time-to-event data [32]. First, the parameter vector is initialized to ${\hat{\beta }}_{0}=\left(0,...,0\right)$. In each boosting step *k *(*k *= 1, ..., *M*) a new candidate model is obtained for every covariate *j *= 1, ..., *p*

(6) $${\hat{\eta }}_{ij,k}={\hat{\eta }}_{i,k-1}+\gamma_{j,k}x_{ij}$$

with the linear predictor from the previous step

(7) $${\hat{\eta }}_{i,k-1}={{\text{x}}_{\text{i}}}^{T},{\hat{\beta }}_{k-1}$$

For obtaining parameter estimates ${\hat{\gamma }}_{j,k}$ a penalized partial log-likelihood is maximized that incorporates a penalty parameter *λ*_*j, k *_which controls the size of the step. The element of the parameter vector ${\hat{\beta }}_{k-1,j*}$, corresponding to that covariate that maximizes the (penalized) log-likelihood is updated by

(8) $${\hat{\beta }}_{k,j*}={\hat{\beta }}_{k-1,j*}+{\hat{\gamma }}_{j*,k}$$

All other elements of the parameter estimation remain unchanged (and therewith zero in most cases). The number of boosting steps *M *is a tuning parameter which needs to be optimized e.g. via cross-validation. Usually, a common penalty parameter *λ *= *λ*_*j, k *_is used for all covariates and boosting steps. It should be chosen in a way the resulting number of boosting steps is larger than 50 [15]. In this study the CoxBoost R-package [33] was used to train the CoxBoost models.

#### Lasso

Lasso [34,35] is a shrinkage method for regression models [[36], chap. 3] with implicit feature selection based on an *L*_1 _penalty term

(9) $$\hat{\beta }=\text{arg}\text{max}\left(l\left(\beta \right)-\alpha ||\beta |{|}_{\text{1}}\right)$$

with a likelihood function *l*(*β*). Originally, quadratic programming was proposed to solve (9) for linear regression models [34]. Since the solution for Cox proportional hazard models is much more computationally intensive, Goeman proposed a solution of the Lasso estimation based on gradient ascent optimization [37]. In this paper the R-package penalized [38] was used to fit the Lasso estimator.

#### Random survival forests

A third method is based on decisions trees. Random survival forests [39] (RSF) is an extension of the Random forests [8] for right censored survival data. A collection of binary decision trees is build by bootstrap samples. In every tree at every node a random subset of *m *features is chosen. The survival difference between the daughter nodes is used to choose a feature and a split point. The R-package randomSurvivalForest [40] was used to train the model. The optimal value of *m *was determined via bootstrap [27,41] using the peperr R-package [28].

### Prostate cancer data set

A prostate cancer data set from Taylor et al. [42] was used in this study. Raw expression data from Affymetrix Human Exon 1.0 ST arrays were obtained from the NCBI GEO data repository (GEO accession number GSE21034) comprising 131 samples of tumor patients. Furthermore, miRNA expression data from the Agilent microRNA V2 were downloaded (GEO accession number GSE21036) including 113 samples of tumor patients.

#### Data preprocessing

Gene expression profiles were derived from the CEL files using Robust Multichip Average (RMA) [43] implemented in the Affymetrix Power Tools (APT). Raw data files from miRNA expression data were analyzed using the limma R-package [44]. After quantile normalization [45] control probes were removed and the 16 replicates of each miRNA were summarized using the sample-wise median. At the end only tumor samples with gene expression as well as miRNA expression data were used yielding a data matrix with 98 tumor samples, 17881 transcripts, and 723 miRNAs.

#### BCR status

Clinical parameters of the patients samples were downloaded from the supplemental material [42]. The time to biochemical relapse (BCR) and the censoring status for 98 cancer patients were available. Of these 98 patients 18 suffered a relapse and 80 were censored.

### miRNA-target predictions

Target predictions were downloaded from MicroCosm targets [21,46] (formerly miRBase Targets) version 5. The p-values of these predictions were extracted for every miRNA-transcript pair (the transcripts were given as Ensembl transcript identifiers). For comparison the TargetScan 5.2 predictions [22] were downloaded.

## Results

### Graph-based integration of miRNA and mRNA expression data

**Figure 2:**
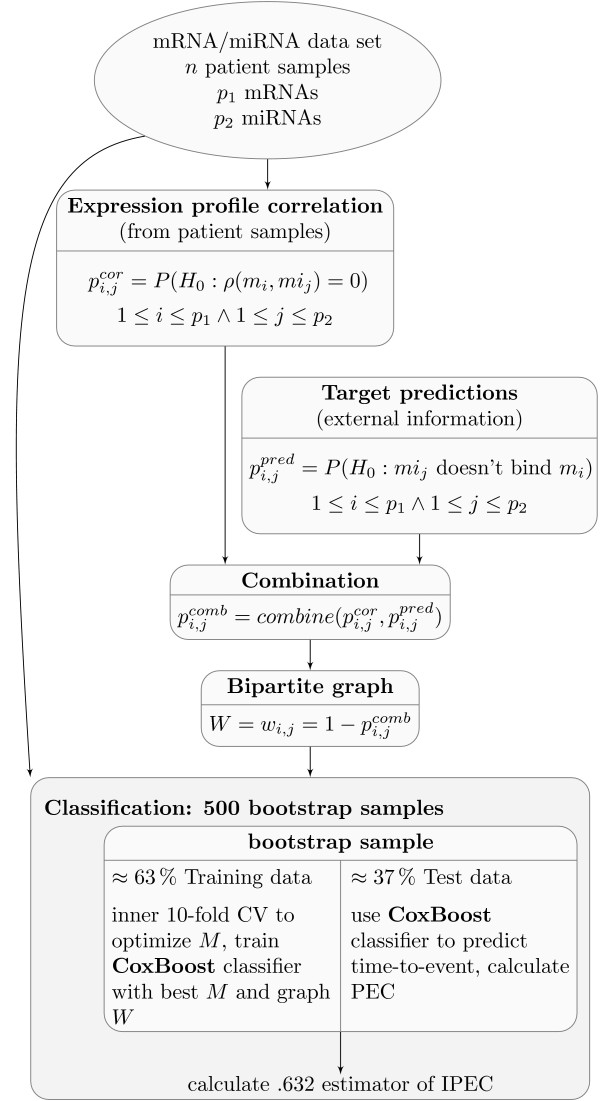
**Workflow Diagram**. Workflow of the integration of miRNA and mRNA expression data.

The first step in the workflow (Figure 2) was the creation of the bipartite graph describing the relations between mRNAs and miRNAs. The first source of knowledge were both expression data sets coming from the same samples. The expression vectors from each mRNA *m_i _*and each miRNA *mi_j _*were correlated using the Pearson correlation *ρ*(*m_i_, mi_j_*). The correlation coefficient can be tested for a significant shift from zero leading to a p-value for every mRNA-miRNA pair

(10) $$\begin{array}{ccc} p_{i,j}^{cor} & =P\left(H_{0}:\rho \left(m_{i},mi_{j}\right)=0\right) & \\ & \forall i\in \left\{1,p_{1}\right\},\;j\in \left\{1,p_{2}\right\} \end{array}$$

Since many tests (*p*_1 _× *p*_2_) were performed, the resulting p-values were corrected for multiple testing [47] (in the following $p_{i,j}^{cor}$ refers to the corrected values). The second source of knowledge were the target predictions from MicroCosm [21]. The p-values $p_{i,j}^{pred}$of these prediction were used to strengthen the importance of the connection of a mRNA *m_i _*and a certain miRNA *mi_j _*in the case where *m_i _*is a predicted target of *mi_j_*. Since the MicroCosm target database holds only mRNA-miRNA pairs with a p-value below 0.05 the p-values of pairs not present in MicroCosm were set to 1.

In order to integrate the two sources of knowledge both types of p-values had to be combined. This was done using the method of Stouffer [48,49] leading to combined p-values

(11) $$\begin{array}{l} p_{i,j}^{comb}=1-\Phi (\frac{1}{\sqrt{2}}(\Phi^{-1}(1-p_{i,j}^{cor})\\ \;\;\;\;\;\;+\Phi^{-1}(1-p_{i,j}^{pred}))) \end{array}$$

where $\Phi \left(x\right)=\int_{-\infty }^{x}\frac{1}{\sqrt{2\pi }}e^{\frac{z^{2}}{2}}dz$ is the probability distribution function of the standard normal distribution. For miRNAs and mRNAs not listed in MicroCosm targets the combined p-values were set to the correlation p-values.

The resulting p-values were well distributed and could easily be transformed to weights

(12) $$w_{i,j}=1-p_{i,j}^{comb}$$

The resulting matrix of weights *W *= *w*_*i, j *_could be viewed as the *p*_1 _× *p*_2 _adjacency matrix of a bipartite graph describing the relations between mRNAs and miRNAs.

The graph *W *was interpreted as a directed graph with edges from mRNAs to miRNAs. In conjunction with CoxBoost the graph was used to improve the prediction of time-to-event data. Binder et al. [15] introduced likelihood-based boosting as a possibility to incorporate gene-gene interaction networks into feature selection in order to improve the prediction performance. The basic idea was to increase the penalty parameter λ_j*_,_l_, l > k after choosing a feature in the *k*-th step

(13) $$\lambda_{j*,l}=\left(\frac{1}{c_{f}}-1\right)I_{j*,l}+\frac{\lambda_{j}^{*},k}{c_{f}}$$

At the same time the penalty of connected features was reduced in the following steps

(14) $$\begin{array}{c} \lambda_{j^{+},k_{m}+1}=\frac{I_{j^{+},k_{m}+1}}{1-\left(1-\frac{I_{j^{+},k_{m}}}{I_{j^{+},k_{m}}+\lambda_{j^{+},k_{m}}}\right)c_{f}}\\ -I_{j^{+}{{,}_{k}}_{{}_{m}}+1} \end{array}$$

$I_{j^{+},k_{m}}$ is the Fisher information in boosting step *k_m _*where the feature was updated the *m_th _*time. This increased the probability of choosing connected features in future steps, leading to feature sets which were consistent with the given a priori information. By what amount the penalty of a selected feature was increased and the penalties of connected features were decreased was determined by a stepsize modification factor *c_f_*.

Similar to graphs describing biological pathway knowledge the mRNA-miRNA graph *W *described the regulations among the features. Every time an mRNA *m_i _*was picked the penalties *λ *of miRNAs connected to *m_i _*were lowered according to the weight of the connection. As a consequence it was more likely to choose a miRNA *mi_j _*highly correlated and being a predicted regulator of *m_i _*in one of the next boosting steps. miRNAs with a connection with high weight to *m_i _*are likely to be a direct regulator of *mi *and therefore of importance for the event as well. The stepsize modification factor was set to a fixed value of 0.9 for all boosting runs.

### Graph information reduces prediction error of CoxBoost

In order to test performance of our new method it was tested using a prostate cancer data set [42] with mRNA and miRNA expression data sets from 98 patients using the biochemical relapse as clinical endpoint. The bipartite graph improved the accuracy of CoxBoost by increasing the probability of selecting miRNAs with connections to already chosen mRNAs (Figure 1). To demonstrate this CoxBoost was trained on both data sets, not given the graph information, and on the single data sets. To assure a comparability of the prediction models a common penalty of 1296 was determined such that the number of boosting steps exceeds 50 in every case (Table 1). The accuracy of the risk prediction models were compared by calculating the .632 estimator of the prediction error curve and its IPEC for 500 bootstrap samples. The medians of the resulting 500 IPECs and their interquartile ranges (IQRs) can be seen in Table 1. To test whether the difference of the IPECs is significant, a one-sided Wilcoxon test was carried out between the single models without a graph and the model incorporating the bipartite graph. It can be seen that CoxBoost performed best when given both data sets and the bipartite graph. For every three risk prediction models without graph information the difference was significant assuming a significance level of 0.05.

**Table 1:** Comparison of Boosting Results.

	M	IPEC (median)	IQR	p-value
**only miRNA**	98	5.90	0.88	*<*0.001
**only mRNA**	100	5.82	0.87	*<*0.001
**both no graph**	99	5.79	0.86	*<*0.001
**both with graph**	99	5.46	1.20	-

The table shows the number of boosting steps *M *for every CoxBoost model and the IPEC (median and IQR) of 500 bootstrap runs. The number of boosting steps were determined using the whole data set. Lower IPEC scores indicate better prediction accuracy. The p-value is the result of a one-sided Wilcoxon test (unpaired) comparing the single data set prediction models and the prediction model without graph with the combination incorporating the bipartite graph.

There was no difference between the models trained only on the mRNAs and the model trained on both data sets without the graph. CoxBoost with only the miRNA expression data seemed to perform slightly worse.

### Comparison with other methods

The CoxBoost model was compared with other methods suited for time-to-event data. The afore introduced Lasso and RSF were trained on the same end point given mRNA data as well as miRNA data. The prediction error was calculated using the same 500 bootstrap samples as before yielding 500 IPECs for every method. Table 2 shows the distribution of the IPECs of Lasso and RSF compared to the IPECs of CoxBoost with graph information. To test the significance of the differences a one-sided Wilcoxon test was used. On this data set Lasso and RSF performed significantly worse than CoxBoost with graph information assuming a significance level of 0.05. Besides the prediction error there was a remarkable difference in the runtime of the three models. Training and prediction for 500 bootstrap samples took 40.17 hours for RSF, 2:25 hours for Lasso, and 1:16 hours for CoxBoost with graph on a 20 core (2.7 GHz) machine with 64 GB memory.

**Table 2:** Comparison with Other Methods.

	IPEC (median)	IQR	p-value
**Lasso**	6.10	1.12	*<*0.001
**RSF**	5.66	0.78	*<*0.001
**CoxBoost with graph**	5.46	1.20	-

The table shows the comparison of Lasso and RSF with CoxBoost with the bipartite graph regarding the prediction error. As before the median and IQR from 500 IPECs were calculated. The p-value is based on a one-sided Wilcoxon test comparing the 500 IPECs of Lasso and RSF with the IPECs of CoxBoost.

### Graph information improves stability of feature selection

In addition to a reduction of the prediction error the incorporation of the graph information improved the stability of the feature selection process remarkably. Table 3 lists the top 10 features of CoxBoost with and without the graph according to the number of bootstrap samples the features were chosen in. The numbers are almost twice as large when including the graph information.

**Table 3:** Selected Features.

No graph		With graph	
Feature	Counts	Feature	Counts
ESM1	161	hsa-miR-513a-3p	329
hsa-miR-412	151	hsa-miR-513a-5p	316
INHBA	130	hsa-miR-128	249
COMP	126	hsa-miR-1226*	233
ZFHX4	114	hsa-miR-1231	209
SLC6A14	103	hsa-miR-1224-5p	206
hsa-miR-484	92	hsa-miR-220a	199
PI15	83	hsa-miR-1233	198
hsa-miR-556-3p	79	hsa-miR-208a	169
hsa-miR-409-3p	74	hsa-miR-199b-3p	168

The table lists the top ten features from CoxBoost with and without graph information. mRNA names are given by their gene symbols (capital letters) while miRNA names are given by their miRBase IDs (starting with *hsa-miR*). The Counts columns indicate in what number of the 500 bootstrap samples the feature was chosen. Consequently, the maximal count would be 500.

Another difference lies in the balance of genes and miRNAs picked by the models. While the number of genes and miRNAs among the top ten features using CoxBoost without graph were almost equal, in the list of CoxBoost with graph information there were only miRNAs.

### Robustness considerations

Additionally, to exclude the possibility of overfitting the models were trained with a graph which was build separately for every single bootstrap sample. Therefore the correlations were calculated and tested solely on the patient samples included in the bootstrap sample. In this case the prediction error increased to 5.64 (median of 500 bootstrap samples) with an IQR of 0.99. In comparison with the IPECs of CoxBoost without graph the prediction error was significant smaller assuming a significance level of 0.05 (p-value from one-sided Wilcoxon test: 0.006). The runtime increased to 21:36 hours.

To asses the influence of the target prediction database one graph was constructed using TargetScan in the version 5.2. As a p-value for a miRNA-mRNA pair 1 - *P_CT _*was used. The *P_CT _*value given in the TargetScan flatfiles is a score that can be used to asses the biological relevance of predicted miRNA-mRNA interactions [22]. 1 - *P_CT _*is an estimate of the FDR. CoxBoost using this graph yielded a median IPEC of 6.60 with an IQR of 0.95.

## Discussion

Due to their role as post-transcriptional regulators of around 30% of the human genome and their involvement in cancer development and progression [17,18,20,50], miRNAs become more and more important for our understanding of the mechanisms leading to cancer. Since miRNAs are smaller than mRNAs they are more stable and in general more resistant against degradation processes than the longer mRNAs. Consequently, miRNA expression is measurable even in serum [51] and paraffin-embedded samples where mRNA expression is hardly detectable.

Several studies have combined gene and miRNA expression data [52,53] or gene expression data with miRNA target predictions [54] to infer new miRNA regulation activities. In addition, several tools have been developed to integrate such data [55,56]. In most cases, correlations between mRNA and miRNA expression profiles gained from matched samples and target prediction scores are most relevant for the analysis.

While there are several approaches to integrate mRNA and miRNA data to discover novel regulatory relation between miRNAs and mRNAs there is still a lack of prediction methods combining both kinds of data into one common prediction model. A central problem in these high-dimensional data is the tendency to overfit. When integrating several *omics *data sets the number of features increases, which makes the feature selection even more important.

In this article we introduce a method capable to fuse mRNA and miRNA expression data in a model to predict a clinical endpoint. Likelihood boosting was used as a method for fitting risk prediction models because of its performance and its ability to implicitly select features in the training process. The correlations between miRNAs and mRNAs and target prediction information were used to model the relations between miRNAs and mRNAs. The combination between these two sources of information was performed on a p-value level using the method from Stouffer [48]. From the combined p-values a bipartite graph could be constructed covering the relations between the two types of features.

The integration of this graph into boosting improves the models in terms of prediction error. In this case the clinical endpoint was the biochemical relapse in prostate cancer using a combined miRNA/mRNA data set of 98 patients [42]. The comparisons of the IPECs clearly showed a significant reduction of the prediction error in comparison with boosting on the single data sets or on the combined data set without the bipartite graph. Here we used the .632 bootstrap estimator of the prediction error because of its simplicity. Other estimators like the .632+ estimator [57] are often used for prediction error estimation for survival models [15,41,58]. It might be less biased but computationally more expensive. First tests with the .632+ estimator lead to comparable results.

Using the graph the feature selection became more stable regarding how often a specific feature was picked in the 500 bootstrap runs. By transferring the weights in the graph from mRNAs to miRNAs, these features were favored. However, it is important to note that miRNA expression data alone failed to predict the relapse as accurate as the combined data with the graph. This may be caused by the fact that one miRNA can have several targets and dysregulation of a miRNA can affect multiple molecular pathways with no direct connection to the outcome. Therefore, the genes as effectors seem to be a mandatory source of information. Among the top 10 features picked using the graph there are some miRNAs found to play a role in prostate cancer, e.g. hsa-miR-128 [59]. However, most of the miRNAs have not been associated with prostate cancer before. It is therefore important to note that it is not straightforward to derive functional implications for single biomarkers from a panel found by a prediction model. The strength of our method is to find miRNA-gene combinations with high predictive power. To investigate whether the selected genes show differences in functional annotations, we also performed a GO enrichment test for the top 100 genes of CoxBoost with and without graph (data not shown). Both sets showed different enriched GO terms. However, no clear patterns concerning cancer related processes occurred.

To assess how our method performed in comparison with other methods suited for time-to-event data, Lasso and RSF were tested on the same data set using the same bootstrap samples. In both cases CoxBoost with the bipartite graph showed a significantly lower prediction error. RSF performed better than Lasso which was worse than CoxBoost without graph on this data set. The runtime of RSF and Lasso was considerably longer than the runtime of CoxBoost with graph on our test system. In this study we used the standard implementations of Lasso and RSF as a reference. As far as we know there are no established ways to combine Lasso or RSF with a graph to guide the feature selection. It might be interesting to see if such methods will improve the prediction error as well. Also other ways of fusing miRNA and mRNA expression data into one model e.g. bundling [60] or kernel based methods [16] have not been considered. Such methods offer a very flexible way of combining different prediction models and might also lead to improvements in terms of prediction error.

To minimize the possibility of overfitting, one CoxBoost model was trained with correlations calculated only on the training data of every bootstrap sample. The resulting prediction error is higher compared to the models with correlations calculated once on the whole data set but it is still significantly lower than CoxBoost with no graph. Further, we showed that the prediction could be improved using the target prediction information from MicroCosm. In order to test the influence of the target prediction database we also tried to incorporate the target predictions from TargetScan. This resulted in a higher prediction error, however. This result can possibly be explained by the lower coverage of TargetScan. From the 723 miRNAs in the data set only 170 could be found in TargetScan having a *P_CT _*value. In comparison, the MicroCosm predictions contained 698 out of the 723 miRNAs with p-values.

While miRNA and mRNA expression data gained from microarray experiments were used in this study, the method is independent of the underlying experimental setup. Next generation sequencing data might be, after the necessary preprocessing steps, used in a similar manner. We presented the fusion of the both data sets with respect to a prognostic time-to-event endpoint. However, in a similar fashion binary endpoints like diagnostic questions or treatment response prediction can be tackled. This would lead to classification problems for which boosting was originally designed and powerful approaches have been formulated. On our setting we would substitute the CoxBoost algorithm by GAMBoost [61].

## Conclusions

With the increasing availability of high-throughput data on many different layers of biological regulation, the integration and fusion of these data sets becomes a key concept when analyzing complex diseases. Combined prediction models involving mRNA and miRNA expression data should include the relations between the different features in the model.

In this article we propose a new method to fuse miRNA and mRNA expression data in a risk prediction model to stratify the risk of a biochemical relapse of prostate cancer patients. In our new approach we combine the CoxBoost model with a bipartite graph assembled from correlations between miRNAs and mRNAs and target prediction information from MicroCosm targets. Using this graph an improvement of the risk prediction could be achieved. Besides an improved risk prediction we could show that the feature selection became more stable and therewith easier to interpret. CoxBoost with graph performed significantly better than two other methods suited for time-to-event data.

The R source code of the proposed method is available in the supplement (see Additional file 1).

## Authors' contributions

SG implemented the method and worked out the examples. SG, HB and TB conceived the method and designed the study. JCB, RK and HS provided the biological background and concept for the study. CP, MF and DW contributed in discussions. All authors contributed to the writing of the manuscript and read and approved the final manuscript.

## Supplementary Material

Additional file 1 **R Code**. The additional file *supp1.r *contains the R functions for the proposed workflow of integrating mRNA and miRNA expression data. Click here for file
